# Study on the Gas Release of 3D-Printed Furan Resin Sand Core during the Casting Process

**DOI:** 10.3390/ma16114152

**Published:** 2023-06-02

**Authors:** Xiaolong Wang, Qihua Wu, Yuhang Huang, Na Li, Xiongzhi Wu, Xiuming Chen, Jiwu Wang, Tao Jing, Tianyou Huang, Jinwu Kang

**Affiliations:** 1 School of Mechanical, Electronic and Control Engineering, Beijing Jiaotong University, Beijing 100044, China; 2 Weichai Power Co., Ltd., Weifang 261061, China; 3 School of Mechanical Engineering, Central South University, Changsha 410000, China; 4 Key Laboratory for Advanced Materials Processing Technology, School of Materials Science and Engineering, Tsinghua University, Beijing 100084, China

**Keywords:** furan resin, sand core gassing, pressure of gas emission, 3D printing

## Abstract

In sand casting, gas porosity is a common defect that can result in decreased strength, leakage, rough surfaces, or other problems. Although the forming mechanism is very complicated, gas release from sand cores is often a significant contributor to the formation of gas porosity defects. Therefore, studying the gas release behavior of sand cores is crucial to solving this problem. Current research on the gas release behavior of sand cores mainly focuses on parameters such as gas permeability and gas generation properties, through experimental measurement and numerical simulation methods. However, accurately reflecting the gas generation situation in the actual casting process is difficult, and there are certain limitations. To achieve the actual casting condition, a sand core was designed and enclosed inside a casting. The core print was extended to the sand mold surface, with two types of core prints: hollow and dense. Pressure and airflow speed sensors were installed on the exposed surface of the core print to investigate the burn-off of the binder of the 3D-printed furan resin quartz sand cores. The experimental results showed that the gas generation rate was high in the initial stage of the burn-off process. The gas pressure quickly reached its peak in the initial stage and then decreased rapidly. The exhaust speed of the dense type of core print was 1 m/s, lasting for 500 s. The pressure peak of the hollow-type sand core was 1.09 kPa, and the exhaust speed peak was 1.89 m/s. The binder can be sufficiently burned off for the location surrounding the casting and the crack-affected area, so the burnt sand appears white, while the burnt core appears black due to insufficient burning of the binder because of isolation from the air. The gas generated by the burnt resin sand in contact with air was 30.7% less than that generated by the burnt resin sand insulated from the air.

## 1. Introduction

**Figure 1:**
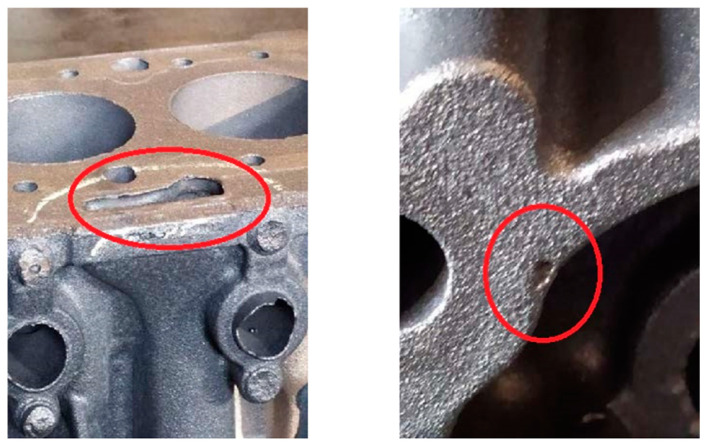
The location of the porosity defects in the engine cylinder block.

In sand casting, the sand core is enveloped by molten metal. When the sand core temperature reaches the decomposition temperature of the resin binder, a large amount of gas is generated from the breakdown of the resin binder. If the gas cannot be discharged in time through the core print, it will invade the unsolidified casting, resulting in casting porosity defects. Gas porosity defects are common in cast iron and aluminum alloy castings. Engine cylinder blocks, cylinder heads, and other castings have many sand cores and complex inner structures. During production, exhaustion is more difficult, which can easily cause gas porosity defects. A typical location of a porosity defect in an engine cylinder block is shown in Figure 1. Porosity defects can cause stress concentration and even fracture, and can also cause problems such as reduced airtightness and fatigue performance of engine castings. Due to the numerous influencing factors and the complexity of the gas porosity formation mechanism, porosity defects have long been a challenging problem.

Considerable experimental research has been conducted on the gas generation of resin cores produced using different processes at different temperatures during the high-temperature decomposition of resin. Bargaoui et al. [1,2,3] studied changes in the quality of resin sand with temperature, and the influence of oxidants on the crushability of resin sand using thermogravimetric analysis. However, this method can only analyze the quality changes of resin sand during the decomposition process, and does not consider the gas volume and pressure. Zhang et al. [4] measured the gas volume of resin sand samples with different resin contents during heating to a specified temperature, using a tube furnace heating method. Under heating conditions of 700 °C to 850 °C, the gas generation rate of a gram of resin sand was 10–15 mL. This method can accurately obtain the gas generation and its rate of resin sand under different temperature conditions, but it significantly differs from the actual casting process, such as the increase in gas porosity caused by core burnout and uneven burnout. Bobrowski et al. [5,6,7] poured gray cast iron and aluminum–silicon alloy melt into the sand mold, with the metal completely enveloping the sand core. They studied the temperature and gas volume changes during the burnout process of furan resin and alcohol–acid resin sand cores by separately extracting the gas produced by the sand core burnout through a duct. The maximum gas emission rate of furan resin sand occurred when the metal in the mold was in the liquid and semi-solid states, but the gas pressure inside the sand core and the effect of the sand core exhaust channel on gas generation were not considered. Walker et al. [8,9] embedded sensors into the mold to collect data such as temperature and pressure at multiple locations to verify the quality of the castings for numerical simulations.

Researchers have also studied the gas porosity defects of castings using numerical simulation methods. Zhang et al. [10] used a conservative phase field-lattice Boltzmann model to analyze the pore defect characteristics of Mg-Al alloys and the variation of bubbles in microchannels with obstacles. Liu et al. [11] simulated the filling and solidification processes of aluminum alloy cylinder heads, found that the porosity defect was caused by the internal sand core being heated to decompose the gas and the gas could not be discharged smoothly, and optimized the structural design to avoid defects. Guofa et al. and Yang et al. [12,13] used numerical simulation analysis to study the casting defects in A356 aluminum alloy wheel hubs during low-pressure casting. They found that internal defects such as shrinkage cavities, shrinkage porosity, and impurity can affect the mechanical properties of the casting. By optimizing process parameters and improving mold design, these defects can be reduced, leading to improved mechanical properties of the casting.

To reveal the actual casting conditions, this study measured the gas pressure and exhaust speed of the sand core during the casting process to investigate the gas evolution law. 3D printing technology was used to produce sand cores with specific structures using furan resin sand [14,15]. The gas in the sand core was designed to be discharged from a single channel. The results contribute to our understanding of the mechanism of gas porosity in castings, and can provide guidance for numerical simulations to predict porosity defects, optimize the casting production process, and reduce porosity defects in castings.

## 2. Experiment

### 2.1. Model Design

**Figure 2:**
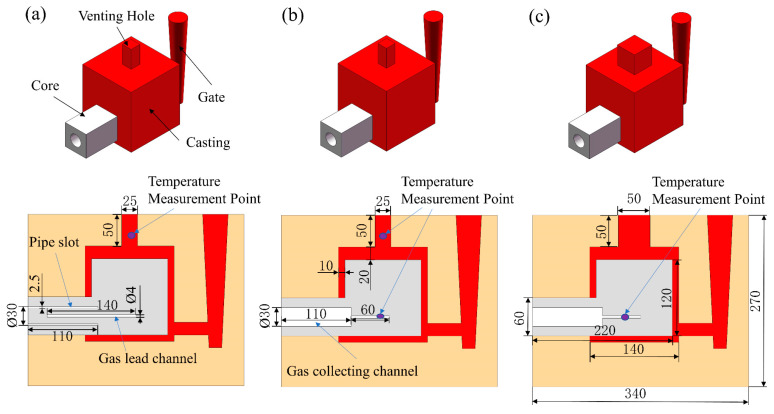
3D model and cross-sections of the casting and sand cores (mm): (**a**) Scheme 1 (with blind core print); (**b**) Scheme 2 (with through-hole core print); (**c**) Scheme 3 (with through-hole core print).

Three experiments were designed to investigate the gas pressure and exhaust speed resulting from the heating and burning of sand cores. The test casting was a 140 mm × 140 mm × 140 mm cube with a 120 mm × 120 mm × 120 mm cubic cavity and a venting hole on the top, as shown in Figure 2. The sand core forms the inner cavity of the casting and is designed with a core print on one side protruding to the surface of the sand mold. During the casting process, the liquid metal can fully wrap around the sand core, and the gas generated by the sand core can escape only from the core print to the environment. This design makes it convenient to measure gas pressure and speed. The size of the sand mold is 270 mm × 340 mm × 270 mm, the size of the sand core is 120 mm × 120 mm × 120 mm, and the core print is 60 mm × 60 mm × 100 mm. The wall thickness of the casting is 10 mm, except for the top 20 mm. To guide the gas to the core print end, an air passage with a diameter of 4 mm was designed in the sand core. Two types of core prints were designed. The first is a blind core print with a circular gap with a diameter of 30 mm; it is 2.5 mm thin and 110 mm deep, and 10 mm of it can penetrate the sand core. It has a tube for gas gathering and a platform for installing the gas speed sensor and pressure sensor. The other is a hollow core print with a central opening with a diameter of 30 mm; it is 110 mm deep, and 10 mm penetrates the sand core. Two sizes of venting holes were designed: one is 25 mm × 25 mm × 50 mm, and the other is 50 mm × 50 mm × 50 mm, resulting in different melt volumes. Their combinations are listed in Table 1.

### 2.2. Sand Molds and Cores Manufacturing

**Figure 3:**
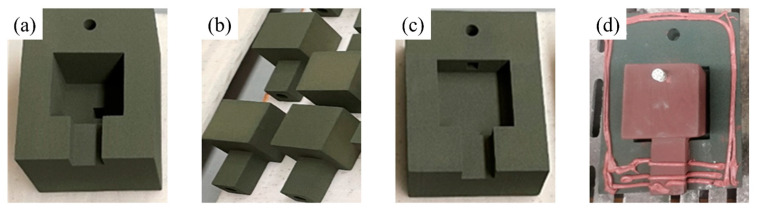
Printed sand molds and sand cores: (**a**) upper sand mold; (**b**) sand cores; (**c**) lower sand mold; (**d**) assembled parts.

The sand molds and sand cores used in the experiment were manufactured using an ExOne S-Max 3D printer. The printing materials were furan resin quartz sand, with a resin addition of 1.5%, and a curing agent addition of 30% of the resin. After printing, the loose sand was removed, and a layer of coating was applied to the surface of the printed sand cores. The sand mold was divided into upper and lower parts for printing, and clay paste was applied to seal the parting surface during assembly. The printed sand mold and sand cores can be seen in Figure 3.

### 2.3. Experimental Design

**Figure 4:**
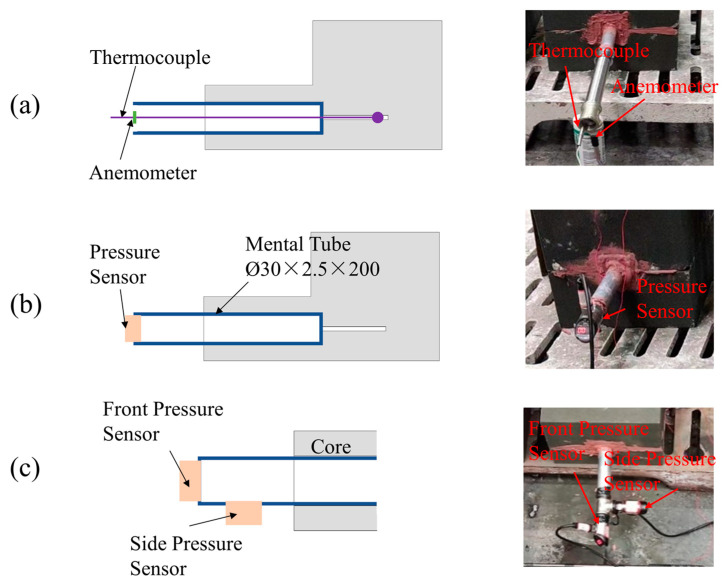
Sensor installation positions: (**a**) thermocouple and anemometer; (**b**) one pressure sensor; (**c**) two pressure sensors.

On-site casting experiments and numerical simulations were performed for the three schemes. Two castings of each scheme were poured. During the experiment, a metal sleeve (a tube 2.5 mm thick) was inserted into the gap of the blind core print and the through-hole of the through-hole core print, as shown in Figure 4. The metal tube completely covered the air lead channel, allowing the gas mainly to escape along the core print. The temperature near the end of the core print was relatively high, and the metal tube helped to cool the exhaust gas, thereby avoiding damage to the gas pressure sensor and anemometer due to the high temperature. A gas pressure sensor (Keyence GP-M001) was screwed to the end of the metal tube and sealed with tape to measure the gas emission pressure. All connection points were sealed. For the hollow core print, a thermosensitive anemometer (SMART AR866A) was inserted at the end of the metal tube to measure the exhaust speed with the sensor blade parallel to the direction of gas flow. Thermocouples were inserted at the top surface of the casting and inside the sand core along the air channel to measure the temperatures of the casting and core. For Scheme 3, gas pressure sensors were installed on the front and side of the metal tube, as shown in Figure 4c. The casting was poured with gray cast iron HT250, and its specific composition is listed in Table 2. The pouring temperature was 1360 °C, and the pouring process lasted for 7 s. In addition, FT GasPore software developed by Tsinghua University was used for the corresponding numerical simulation and further analysis, with version 1.0.0.

The parameters measured in the experiment include casting temperature, sand core temperature, gas emission pressure, and gas emission speed. High-temperature S-type thermocouples were used to measure the casting temperature, while K-type thermocouples were used to measure the sand core temperature. The sampling frequency for temperature, pressure, and speed was 1 Hz, and data collection was performed using a GRAPHTEC GL7000 data logger. The measurement items are shown in Table 3, with *T_cast_* representing the casting temperature, *T_core_* representing the sand core temperature, and *v* representing the gas emission speed at the sand core print. *P_front_* represents the gas emission pressure on the front of the metal tube at the sand core print, while *P_side_* represents the gas emission pressure on the side wall.

## 3. Results

### 3.1. Casting and Sand Core Temperature

**Figure 5:**
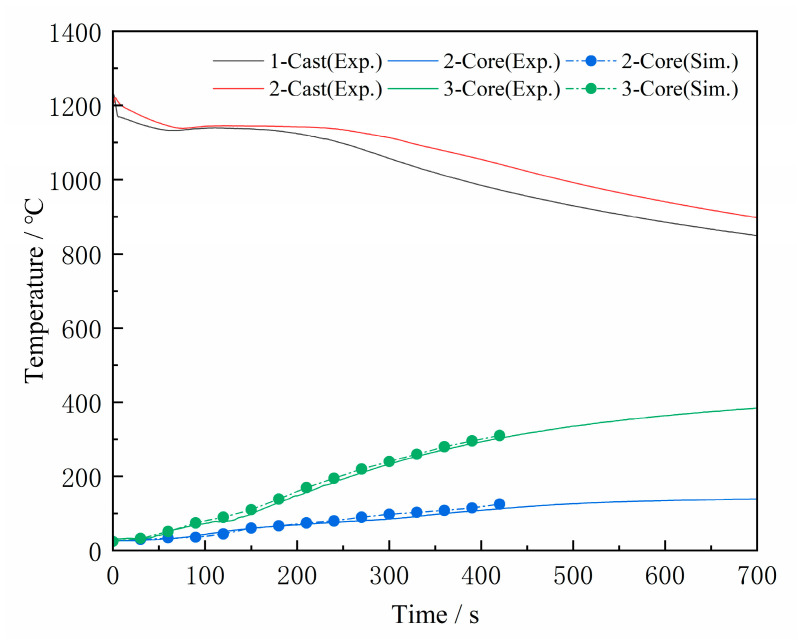
Temperature variation curves of casting and sand core.

Figure 5 shows the temperature change curves of the casting and sand cores, with time 0 representing the start of pouring. Due to unavoidable heat loss during iron transportation and pouring, the temperature peak of the casting is around 1240 °C after pouring, before it gradually decreases. The temperature change trends of all castings are similar. The temperature inside the sand core slowly increases. After 700 s, the sand core in Scheme 2 reached 150 °C, while the sand core in Scheme 3 reached 400 °C. The position of the thermocouple in Scheme 3 is not the same as in Scheme 2 due to an error during thermocouple insertion. The temperature measurement position of Scheme 3 is closer to the outer surface of the sand core, resulting in a higher temperature compared to Scheme 2. This can be proven using numerically simulated cooling curves.

### 3.2. Gas Pressure

**Figure 6:**
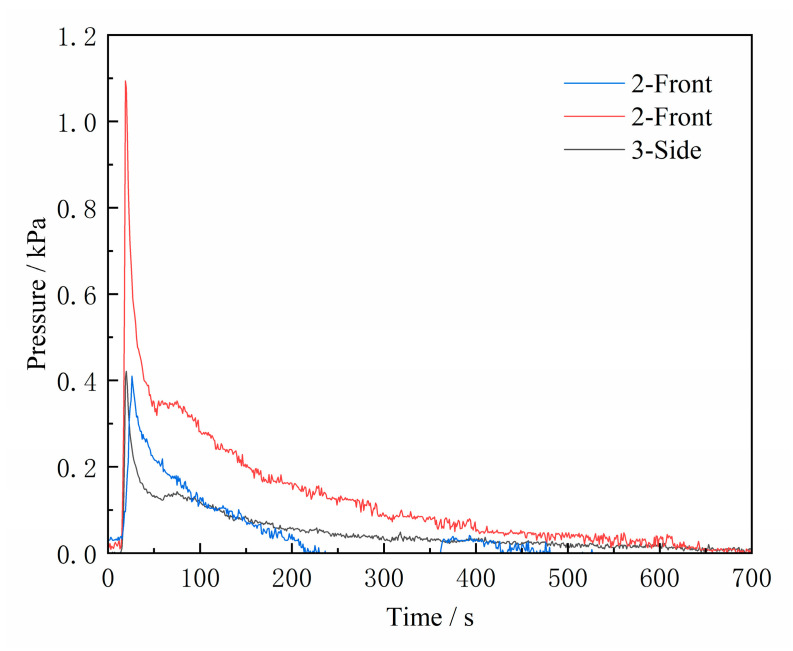
Gas pressure variation curves.

**Figure 7:**
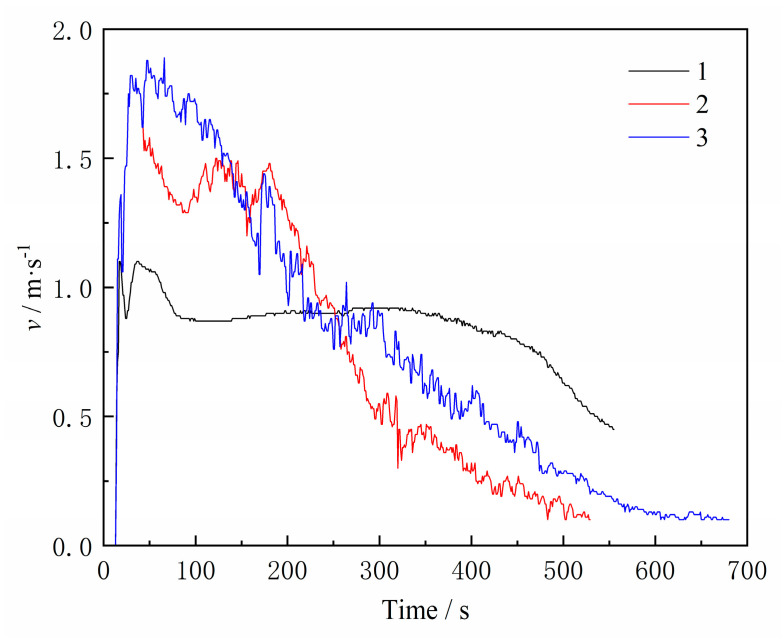
Exhaust speed change curves.

Figure 6 compares the gas pressure at the core print of the sand cores cast with different amounts of molten iron. The gas pressure rapidly increased and reached its peak when the pouring was just completed. The peak values for Schemes 2 and 3 are 0.41 kPa and 1.09 kPa, respectively. As can be seen by comparing the temperature curves in Figure 7, gas generation in the sand cores mainly occurs in the initial stage. During this stage, a large amount of gas is generated due to the high-temperature decomposition of the binder on the surface of the sand cores, in direct contact with the molten metal. After the filling process is completed, the gas release gradually stabilizes. Following the peak, the pressure drops at a relatively fast rate for a certain period due to the increased porosity of the sand particles resulting from the decomposition of the binder at high temperatures, which allows gas to enter the gap and causes the pressure to drop.

Increasing the amount of molten iron for the same type of sand core structure increases the degree of burning of the sand cores, leading to an increase in the peak gas pressure inside the sand cores. Under the same test conditions, the gas pressure measured at different locations differs significantly. The experiment measured the gas pressure on the front and side surfaces of the metal tube in Scheme 3, and the peak pressure on the side was only 0.42 kPa. This is because the gas is dynamically discharged from the interior, and there is a certain inertia during the flow, which will cause a certain impact force on the sensor located at the front of the core print, resulting in a higher gas pressure on the front than on the side. This case is different from a closed space in a static condition, wherein the pressure is the same at any location and direction.

### 3.3. Exhaust Speed

Figure 7 compares the air speed at the core print of three types of sand cores. At 12 s, the gas speed sharply increased, indicating that a large amount of gas was generated inside the core at the beginning of the pouring, which is consistent with the trend of gas pressure changes. Then, the exhaust speed gradually decreased. Both Scheme 2 and Scheme 3 have a hollow sand core structure, and their air speed changes are the same. After the filling was completed, the exhaust speed quickly reaches peaks of 1.82 m/s and 1.89 m/s, respectively, and then gradually decreases. In contrast, for Scheme 1 with a dense core print structure, its peak air speed is lower than the other two groups, with a value of 1.10 m/s due to the obstruction of the sand. However, the exhaust speed was sustained at about 1 m/s for approximately 500 s. This indicates that the reasonable arrangement of exhaust channels in actual casting processes is conducive to the rapid discharge of gas generated inside the core. The circular tube section area is 3.14 cm^2^ and the effective section area of the outlet is 2.14 cm^2^. The total gas generation of the three groups is obtained using Equation (1). The sand-to-mental ratios, pressure peaks, speed peaks, and measured total gas generation of the three groups are shown in Table 4, where the sand-to-mental ratio refers to the ratio of the weight of the sand core to that of the casting (excluding the core print). In addition, here, the gas is heated and at a high temperature when released, and the volume will expand by about three to four times compared to room temperature. Scheme 3 has the smallest sand-to-metal ratio and therefore the largest total gas generation volume.

(1) V=S∫vdt

where V is total gas, S is area of exhaust outlet, and v is speed.

## 4. Discussion

### 4.1. Heating up of Core and Binder Burning

**Figure 8:**
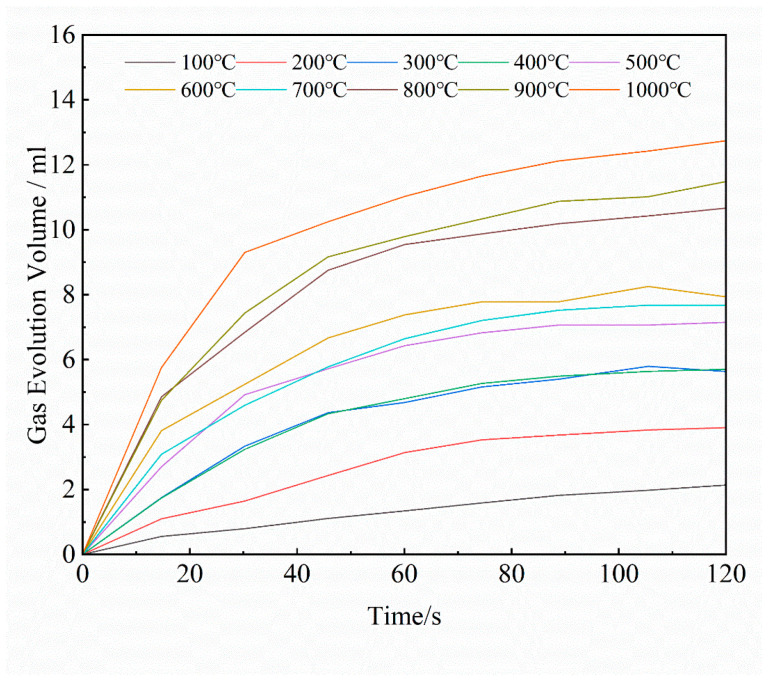
Gas release curve of 1 g furan resin-bonded sand due to heat decomposition.

**Figure 9:**
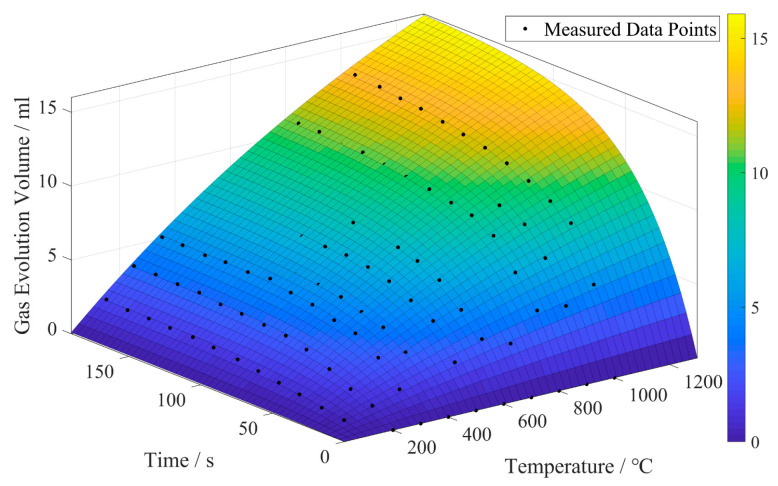
The fitting surface of gas release during thermal decomposition of 1 g furan resin sand.

**Figure 10:**
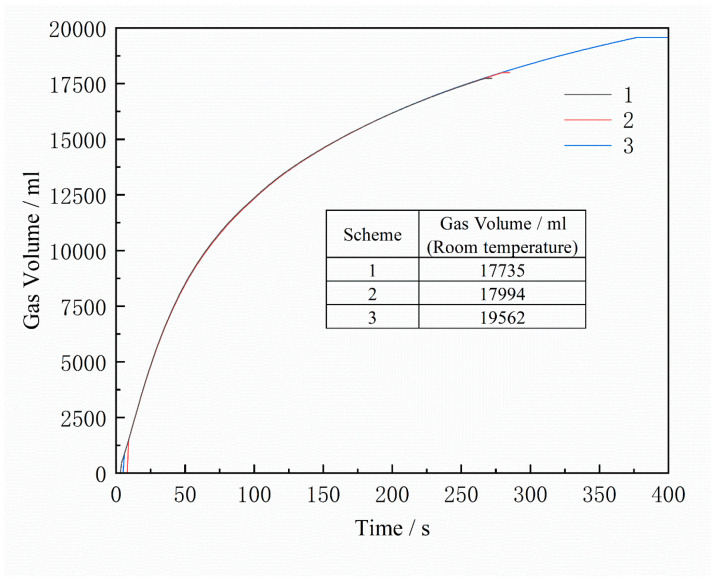
Simulated gas release curve of the sand core.

The gas release of the core during heating was measured using the Beijing Jia Tian Modeling Material Testing Workstation. Curves of 1 g furan resin-coated quartz sand heated for 120 s at different temperatures are shown in Figure 8. It is notable that the total gas evolution increases with temperature and time. During the binder decomposition process in the initial 0–40 s at the same temperature, the gas release rate is higher, and stabilizes after heating for 100–120 s. By fitting the curves using MATLAB, the gas release surface of the furan resin sand concerning temperature and time is obtained, as shown in Figure 9. Using this fitting result and Formula (2), the total gas evolution curve of the sand core during the casting process is calculated, as shown in Figure 10. Scheme 3 has the largest sand-to-metal ratio, so the total gas generation volume is the largest. Scheme 2 and Scheme 1 have the same sand-to-metal ratio, but there is a difference in gas volume due to the difference in core head structure; however, this difference is small and negligible.

(2) $$V=\sum_{n=1}^{N}\sum_{T_{0}}^{T}\sum_{t}^{\Delta t}f(T,t)$$

where *V* is the total gas release, *N* is the number of mesh elements used in the numerical simulation, *T* is temperature, and *t* is time, meaning f(T,t) refers to the gas release function.

**Figure 11:**
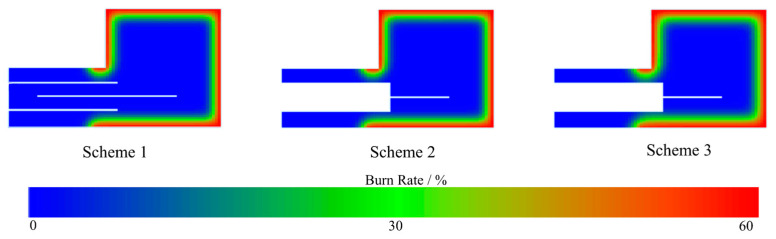
Cross-sectional view of sand core burn-off after 2.5 min of pouring.

**Figure 12:**
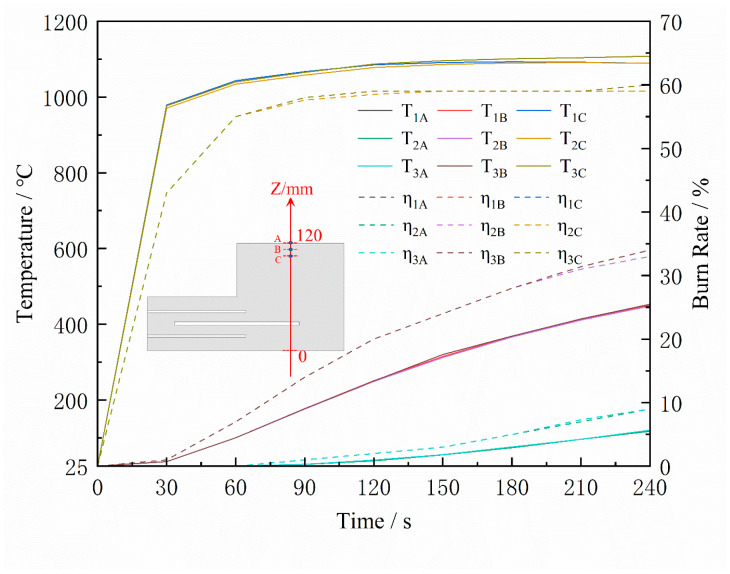
Temperature (T) and burn-off rate (η) curves at points A, B, and C of the sand core.

The simulated burn-off rates of the sand cores after pouring for 150 s are shown in Figure 11. The burn-off rates of the three schemes are mainly concentrated on the surface in contact with the casting, and the burn-off rate of the surface reaches 60%, while the internal burn-off degree is relatively low. The depth of the burn-off at the top of the sand core is greater in Scheme 3 due to the larger venting pin size. Figure 12 shows the temperature and burn-off rate variation curves at points A, B, and C on the central cross-section of the three sand cores (Point A is located on the surface, and the distance between the points is 10 mm). It can be seen that the burn-off rate of all three schemes rapidly reached 45% after 30 s of casting, and stabilized at 60% after 60 s, which is much higher than the burn-off rate of Points B and C. The temperature at Point A was much higher than that of Points B and C. Only a thin layer close to the surface of the core is heated up and burned, and the main part of the core is still at a low temperature, with zero binders burning off. From Figure 11, it can be seen that the main gas release is from the surface of the core in contact with the casting, so the gas pressure and the exhaust speed peak rapidly within 0–60 s after pouring. Therefore, the gas pore probably occurs in this period.

**Figure 13:**
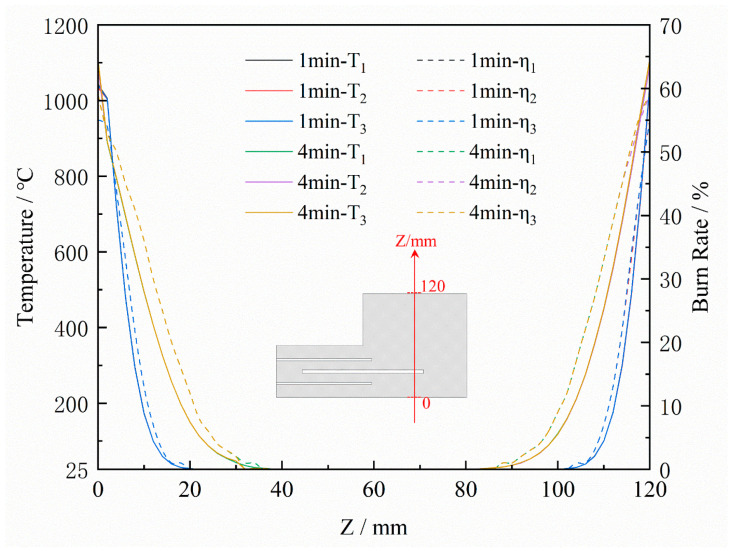
Variation curves of sand core temperature and burnout in the Z-direction of the central section at 1 min and 4 min.

Figure 13 shows the temperature and burnout distribution on the Z-directional center section of the sand core. The maximum burn-off thickness of the sand core was 20 mm after 1 min and 40 mm after 4 min. The temperature of the sand core varied rapidly in the Z-direction, with high surface temperatures and low central temperatures. The heating and burnout of the sand core’s core region were slow.

### 4.2. Binder Burning Mechanism

**Figure 14:**
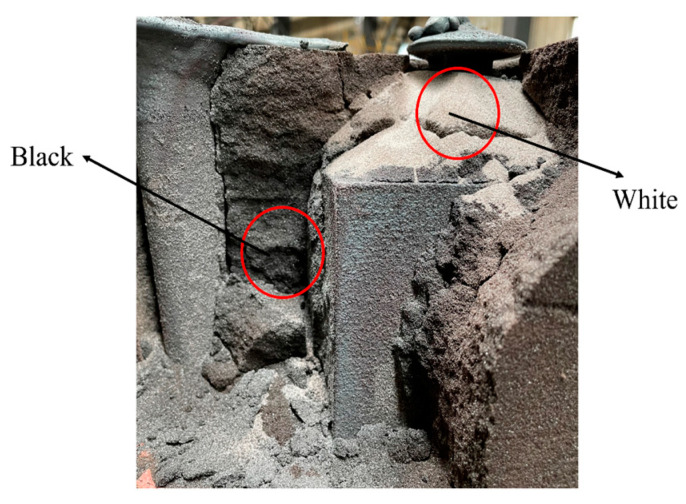
Resin sand burnout situation.

Figure 14 shows the burned sand mold and core. The sand around the venting channel turned loose and white, the core and broken mold stayed close to the contact surface, and the casting kept the original shape and appeared black with clear burning traces. This indicates that the sand in contact with the casting may have undergone the same heating history, but the binder burning is different.

**Figure 15:**
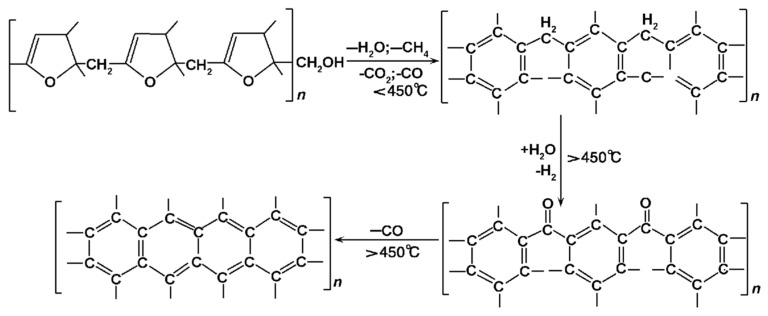
Thermal cracking of furfuryl resin at high temperature (−: generation; +: consumption) Reprinted with permission from Ref. [20]. 2009, Jigang Wang.

**Figure 16:**
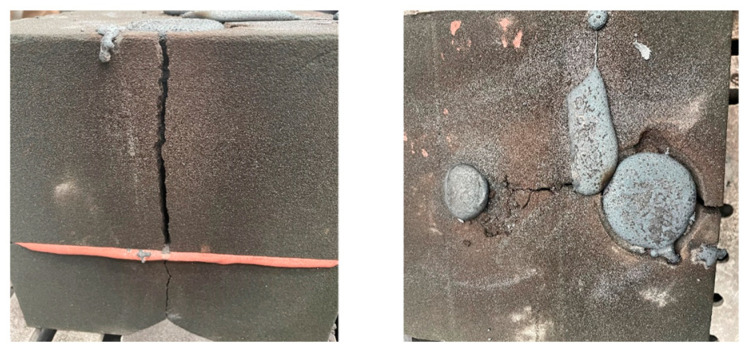
Cracks on the sand mold.

The furan resin-bonded quartz sand has a density of 1.6 g/cm^3^. Furan resin is an organic synthetic resin with a stable furan ring structure, which results in a higher residual carbon content after carbonization due to its high oxygen content. When treated at 900 °C in a closed space, the residual carbon content is 54.9%. The chemical changes that occur during the high-temperature pyrolysis of furan resin are shown in Figure 15, and the generated gases include H_2_O, CO_2_, CO, H_2_, and CH_4_ [16,17,18,19]. During the experiment, the sand core was wrapped in molten iron, making it impossible to contact the external air. Only a small amount of air remained in the pores of the sand core, as the core has a porosity of roughly 40%. The oxygen is only around one-fifth of the entrapped air, so the oxygen is around 8% of the core volume. It can be inferred from Table 5 that the amount of residual air was approximately 0.08 mL in a 1 cm^3^ sand cube, which is negligible compared to the gas release volume. Therefore, a portion of the sand core pyrolysis residue inside the casting remains as amorphous carbon and cannot be fully burned, resulting in blackening after combustion. As the sand mold around the venting channel cracked during the casting process, air penetrated the mold along the crack, as shown in Figure 16. The gases and amorphous carbon produced due to the pyrolysis of furan resin can undergo enough oxidation–reduction reactions with O_2_ to generate CO_2_ and H_2_O, resulting in the full burning of residual carbon after combustion. Finally, there is neither binder nor residual carbon, and the sand turns loose and white.

Due to the limited research available on the complex process of thermal decomposition of furan resin, phenolic resin is used as a reference to analyze the effect of oxygen on the gas produced from the sand core’s decomposition. Under high-temperature anaerobic conditions, the thermal decomposition products of the phenolic resin include volatiles such as H_2_O, CO_2_, CO, CH_4_, C_2_H_6_, H_2_, phenol, methyl derivatives, and some condensed hydrocarbons, as well as non-volatile amorphous carbon. After being subjected to 1000 °C heat treatment, the residual carbon yield is 60.5% [21]. The simplified types and percentages of phenolic resin thermal decomposition products are shown in Table 6, among which CH_4_, H_2_, and CO are the main gases that can react with O_2_ [22]. According to the oxidation–reduction reaction equation in Table 6, the total gas volume after the reaction decreases by 0.307 mol (including the air involved in the reaction). During the combustion reaction of non-volatile amorphous carbon, C + O_2_ = CO_2_, the gas volume remains unchanged before and after the reaction. There is very little entrapped oxygen inside the sand core, so the amount of gas reduction before and after the reaction is determined by the amount of oxygen, as shown in Table 7. The gas is reduced by only 0.03% after the reaction, which is negligible. When measuring the gas generation volume in the test, the average temperature of the gas in the flow through the anemometer is about 900 °C. The volume will expand about 3.9 times, and the volume at room temperature is shown in Table 8. It is still higher than the simulated gas volume at room temperature, as the simulation results are about 70% of the measured results. This is mainly because the gas generation volume per gram of sand used in the simulation calculation is measured under the condition of sufficient oxygen, and the sand core, due to the casting encapsulation, is in an oxygen-deficient environment (which is why this error occurs). From Figure 10, we can observe that the total volume of gas generation in Scheme 1 and Scheme 2 is almost the same, while in Table 4, the measured volume of gas generation in Scheme 1 is higher than that in Scheme 2. This is because the core print of Scheme 2 is a through-hole structure, and part of the sand core is in contact with the oxygen in the air, which generates less gas than the blind hole-type core print structure in Scheme 1 under the same sand-to-metal ratio. Additionally, in the numerical simulation, the gas release model is based on traditionally measured results, as shown in Figure 9. Therefore, the simulated results did not include the effect of the reaction of gas products with the oxygen in the air.

In conclusion, with the same degree of burning, the gas produced by the sand core is 30.7% less than that produced by the sand core in the casting due to contact with air, and the color after burning is white. Therefore, experiments testing sand gassing should consider isolating the air for more accuracy.

## 5. Conclusions

Gas release and exhaust tests were conducted on sand cores with two different types of core prints: blind and hollow. The tests measured the temperature, gas pressure, and exhaust speed of both the casting and sand cores. The following conclusions were drawn with a casting–core ratio of around 2.8:

1. The rate of gas generation in the furan resin sand core was high during the first 60 s after pouring, reaching a gas pressure peak of 1.09 kPa.
2. The gas release of sand cores lasted for 10 min. The sand core with a blind core print structure had a peak speed of 1.10 m/s, and maintained a constant exhaust speed of 1 m/s for 500 s. The exhaust speed of the sand core with a hollow structure quickly reached a peak of 1.89 m/s and then slowly decreased.
3. There is the possibility of gas pores occurring during the initial stage of casting due to an eruption of gas release.
4. Since the sand core was wrapped in molten metal, the air gaps inside the core were unable to fully support the combustion of the products of the pyrolysis of the binder. After the binder was burnt, residual carbon accumulated, resulting in blackened internal resin sand. However, on the surface or area with cracks, the burnt surface resin sand turns white. The gas volume generated by the burnt resin sand under anaerobic conditions is 30.7% higher than that in contact with air. Therefore, there is more gas released in the core than the amount found using a calculation based on a gas evolution test of core sand, which is carried out in conditions with enough air. Traditional gas evolution testing of mold or core sand should be modified to the state of the sand sample so that it may be insulated from the air.

## Figures and Tables

**Table 1:** Designed schemes of casting and sand core models.

Scheme	Core Print Structure	Venting Hole/mm
1	Blind hole	25 × 25 × 50
2	Through-hole	25 × 25 × 50
3	Through-hole	50 × 50 × 50

**Table 2:** Chemical composition of the gray cast iron HT250 (wt%).

Element	C	Si	Mn	P	S	Fe
Content	3.12	1.56	0.9	0.1	0.12	Balance

**Table 3:** Experimental test plans.

Scheme	Core Print Structure	Measurement Items	
		No. 1	No. 2
1	Blind hole	*T_cast_ v*	/
2	Through-hole	*T_cast_ T_core_ v*	*P_front_*
3	Through-hole	*T_core_ v*	*P_front_ P_sider_*

**Table 4:** Test process measurement.

Scheme	Core Print Structure	Sand-to-Metal Ratio	Peak Pressure/kPa	Peak Speed/m·s^−1^	Total Gas/mL
1	Blind hole	0.284	/	1.10	98,265
2	Through-hole	0.284	0.41	1.82	89,700
3	Through-hole	0.266	1.09	1.89	108,776

**Table 5:** Oxygen content and pyrolysis gas volume of 1 cm^3^ sand core.

Name	Unit	Value
Sand	cm^3^	1
Porosity	/	40%
Density	g/cm^3^	1.6
Entrapped air	mL	0.4
Entrapped O_2 in_	mL	0.08
Pyrolysis gas	mL	25.6

**Table 6:** Types and percentages of pyrolysis gases produced by phenolic resin.

Gas Type	Gas Content/mol [22]	Redox Reaction Equation	Volume Change/mol
H_2_O	0.258	/	0
CH_4_	0.11	CH_4_ + 2O_2_ = 2H_2_O + CO_2_	0
CO	0.063	2CO + O_2_ = 2CO_2_	−0.0315
H_2_	0.551	2H_2_ + O_2_ = 2H_2_O	−0.2755
CO_2_	0.018	/	0
Sum	1		−0.307

**Table 7:** Types and percentages of pyrolysis gases produced by phenolic resin in entrapped conditions.

Gas Type	Gas Content/mol	Entrapped O_2_/mol	Redox Reaction Equation	Volume Change/mol
H_2_O	0.258		/	0
CH_4_	0.11		/	0
CO	0.063	0.003	2CO + O_2_ = 2CO_2_	−0.003
H_2_	0.551		/	0
CO_2_	0.018		/	0
Sum	1	0.003		−0.003

**Table 8:** Simulated gas volume and tested gas volume.

Scheme	Simulated Gas Volume/mL (Room Temperature)	Tested Gas Volume/mL	
		900 °C	Room Temperature (25 °C)
1	17,735	98,265	24,966
2	17,994	89,700	22,789
3	19,562	108,776	27,636

## Data Availability

Not applicable.
